# Non-linear relationship between body roundness index and albuminuria among children and adolescents aged 8–19 years: A cross-sectional study

**DOI:** 10.1371/journal.pone.0299509

**Published:** 2024-03-07

**Authors:** Xuankai Qin, Jiahui Wei, Jie Chen, Fengying Lei, Yuanhan Qin

**Affiliations:** Department of Pediatrics, The First Affiliated Hospital of Guangxi Medical University, Nanning, Guangxi, China; Tehran University of Medical Sciences, ISLAMIC REPUBLIC OF IRAN

## Abstract

**Introduction:**

Obesity has been found to be correlated with numerous health issues, including an elevated risk of albuminuria in adults. However, this correlation is still controversial among children and adolescents, as several recent large-scale cross-sectional studies have observed a negative correlation between obesity and albuminuria. Our study aimed to investigate the link between the body roundness index (BRI) and albuminuria among children and adolescents, in order to further understand the correlation between obesity and albuminuria in this demographic.

**Methods:**

We employed information from the National Health and Nutrition Examination Survey (NHANES) 1999–2010 for cross-sectional analysis. Weighted logistic regression was employed to explore the linear relationship between BRI and albuminuria, with subgroup analyses performed for more detailed insights. Weighted linear regression analysis was employed to explore the relationship between BRI and the urine albumin-creatinine ratio (UACR). Additionally, we applied smooth curve fitting to investigate their non-linear relationship and conducted threshold effect analysis to identify any turning point.

**Results:**

In this study of 15,487 participants aged 8–19 years, multivariate logistic regression analysis revealed a significant negative correlation between BRI and albuminuria (OR = 0.616, 95%CI: 0.526–0.722). The relationship between BRI and UACR, as shown by multivariate linear regression analysis, was significantly inversely correlated (β: -5.424, 95%CI: -7.416 to -3.433). Furthermore, smooth curve fitting and threshold effect analysis showed a non-linear relationship between BRI and albuminuria, with a BRI inflection point identified at 2.906.

**Conclusions:**

These findings of our study suggest a significant nonlinear negative association between BRI and the presence of albuminuria among children and teenagers, and maintaining an appropriate BRI may decrease the occurrence of albuminuria in this population.

## Introduction

Childhood and adolescent obesity is on an upward trend, with severe obesity increasing fourfold within the past 35 years [1]. This trend is alarming due to the established link between obesity and multiple health issues like diabetes, hypertension, and metabolic syndrome [2,3].

Albuminuria, characterized by an excessive amount of albumin in the urine, can occur when kidneys are damaged and is indicative of kidney diseases such as chronic kidney disease (CKD) [4,5]. Past research has also shown a connection between albuminuria and an elevated risk of cardiovascular disease [6,7].

Past research on adults has established a positive correlation between obesity and albuminuria [8–11]. Mechanistically, obesity can lead to the activation of the sympathetic nervous system and the renin-angiotensin system, resulting in elevated blood pressure and hyperperfusion of the glomeruli [12]. Additionally, obesity can trigger the release of adipokines, leading to chronic inflammation. These factors can damage the glomerular filtration barrier, leading to the occurrence of albuminuria. Furthermore, recent research has shown that when adipose tissue cannot accommodate excess lipids, these lipids would overflow into the circulation and subsequently accumulate in mesangial cells and renal tubules, amplifying renal injury [13].

Nonetheless, among children and adolescents, research on the correlation between obesity and albuminuria is limited and controversial [14]. Contrary to the positive link observed in adults, several large-scale cross-sectional studies have actually revealed a negative correlation between body mass index (BMI) and albuminuria among children and adolescents [15,16]. Hence, given the increasing prevalence of obesity and conflicting research results, a thorough understanding of the link between obesity and albuminuria in children and adolescents is crucial for promoting kidney health.

While BMI is extensively utilized in obesity research, it possesses constraints, such as the incapacity to discern between the mass of muscle and fat [17]. The body roundness index (BRI), an innovative obesity-related human body measurement indicator put forward in 2013, offers a more precise estimation of body fat and visceral fat than BMI [18]. A higher BRI corresponds to more body fat accumulation and a rounder physique. Previous research has demonstrated that in children and adolescents, BRI better predicts cardiac metabolic risk factors, including hypertension, dyslipidemia, and abdominal obesity, compared to BMI [19]. The relationship between higher BRI and lower eGFR was observed in a cross-sectional study that included 36,784 adults [20]. However, to date, no research has been conducted on the correlation between the BRI and albuminuria among children and adolescents. Hence, utilizing data from the National Health and Nutrition Examination Survey (NHANES) collected between the years 1999–2010, we performed a cross-sectional study to explore the link between BRI and albuminuria in this demographic, aiming to further comprehend the connection between obesity and albuminuria among youngsters. Furthermore, smooth curve fitting was employed to investigate whether a nonlinear association exists.

## Methods

### Data source

NHANES, designed and implemented by the National Center for Health Statistics (NCHS), is a cross-sectional survey that collects information on the health and nutrition status of people in the United States, with data being released in two-year cycles.

### Ethics statement

Approval for NHANES was granted by the Ethics Review Board of NCHS, and written consent was secured from all participants. The database ensures the anonymity of participants. Additionally, the database is accessible to researchers without application, and no additional Institutional Review Board approval is required for data analysis.

### Study population

**Fig 1:**
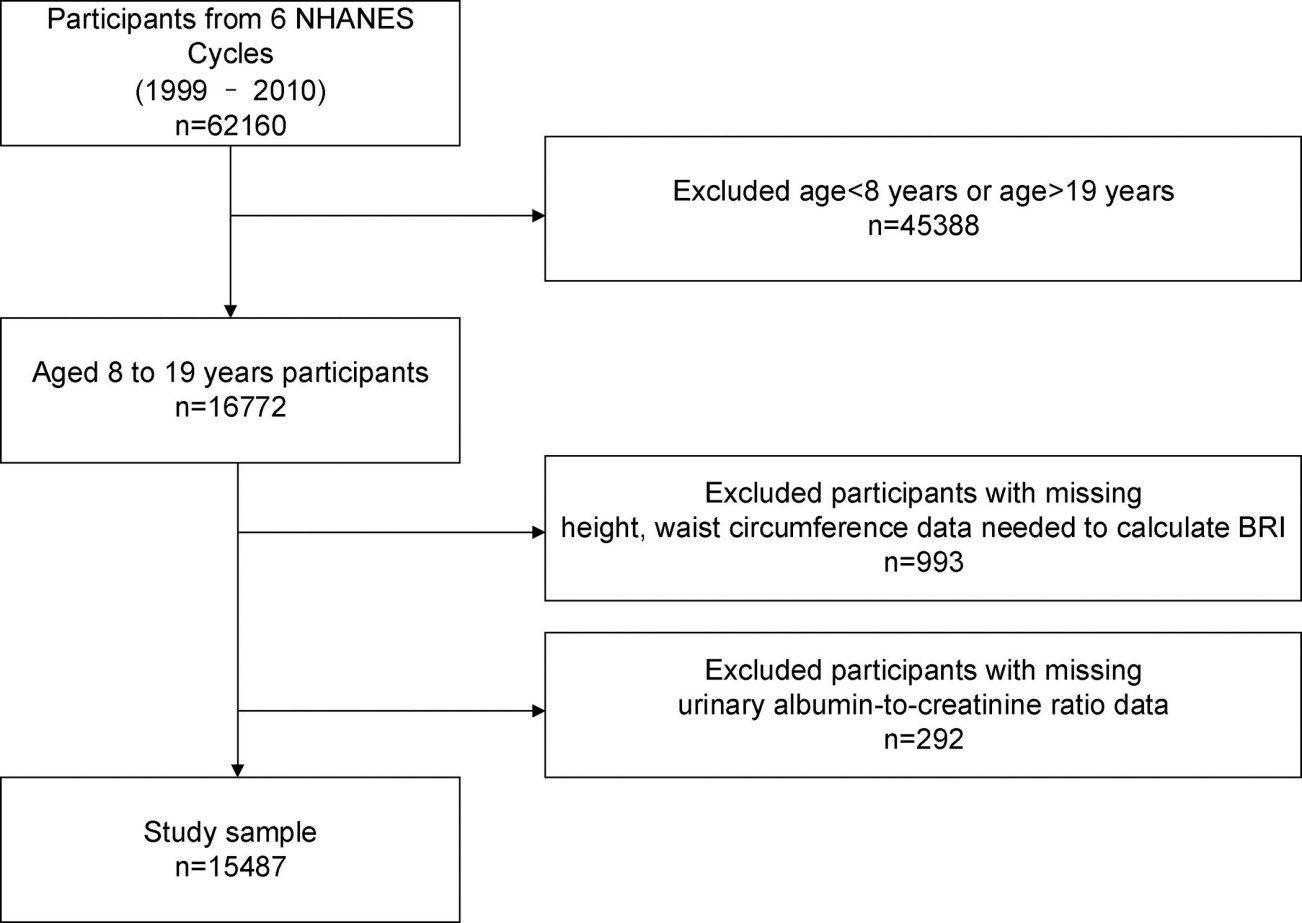
The flowchart of participants.

Our study was conducted using data from the years 1999–2010 and initially included 16,772 participants between the ages of 8 and 19. From these participants, we further excluded 993 individuals due to missing height and waist circumference data needed to calculate BRI, as well as 292 who were missing urine albumin-creatinine ratio (UACR) data. Ultimately, our study included 15,487 individuals. The detailed selection process is presented in Fig 1.

### Exposure variable

Our study employed BRI as the exposure variable. Standardized examination protocols were adhered to by trained NHANES health technicians and recorders for the accurate measurement and recording of anthropometric measurement data. BRI was determined by taking into account the height (in m) and waist circumference (in m) [17]. The formula for calculation is as follows:

$$BRI=364.2-365.5\times \sqrt{1-{\left(\frac{Waist\;Circumference}{2\pi }\right)}^{2}/{\left(\frac{Height}{2}\right)}^{2}}$$

### Outcome variables

The study utilized albuminuria and UACR as the outcome variables. With its efficiency and convenience, the UACR has been extensively used for the evaluation and definition of albuminuria [21]. In our study, we considered a UACR greater than 30 mg/g as indicative of albuminuria [22].

### Covariates

With the aim of controlling for potential confounding variables, variables that were referenced in prior studies or that led to an effect value change exceeding 10% upon their inclusion in the model were considered as covariates. These covariates consist of: demographic characteristics such as age, gender, and race; physical examination findings such as systolic and diastolic blood pressure; laboratory examinations including alanine aminotransferase (ALT), aspartate aminotransferase (AST), glycated hemoglobin, estimated glomerular filtration rate (eGFR), low–density lipoprotein cholesterol, high–density lipoprotein cholesterol, total cholesterol and C-reactive protein [20,23]. Using the creatinine equation from the Chronic Kidney Disease Epidemiology Collaboration, the eGFR was calculated [24].

### Statistical analyses

Statistical analysis was performed using suitable NHANES sampling weights, considering the complex multi-stage cluster survey. All analyses were performed using R (version 4.1.3) and EmpowerStats software (http://www.empowerstats.com). Values of BRI that were either below the 1st percentile or above the 99th percentile were considered extreme values and were excluded from the analysis. When analyzing the baseline characteristics, continuous variables were analyzed with the weighted student’s t-test and reported as mean (standard error) to reflect the complex survey design; while categorical variables expressed as percentages, were examined employing the weighted chi-square test. Subsequently, weighted multivariate logistic regression analysis was employed to investigate the association between BRI and albuminuria. Covariates were gradually adjusted through Models 1 to 3: Model 1 is unadjusted, Model 2 makes adjustments for age, gender, and race, and Model 3 adjusts for all covariates. We further conducted subgroup analyses by gender, age, and ethnicity to determine the link among different subgroups. Weighted multivariate linear regression analysis was employed to explore the association between BRI and UACR. Moreover, we carried out smooth curve fitting and utilized threshold effect analysis model to explore the nonlinear correlation between BRI and albuminuria, and to identify any potential inflection point.

## Results

### Baseline characteristics

Table 1 displays the detailed weighted baseline characteristics of the study participants. The study population had a mean age of 13.489 years, with 50.797% being male and 49.203% female. Among them, 12.159% were diagnosed with albuminuria, and the average BRI was 3.229. Based on their BRI levels, we categorized the participants into four quartile groups. Participants from the highest BRI quartile were found to be older, had a lower incidence of albuminuria and higher levels for height, waist circumference, systolic and diastolic blood pressure, ALT levels, low-density cholesterol, total cholesterol, C-reactive protein and glycated hemoglobin when compared to those from the lowest quartile. On the other hand, their eGFR, UACR, AST and high-density cholesterol levels were lower.

**Table 1:** Basic characteristics of participants categorized by BRI quartile.

Characteristics	Q1 < 2.145	2.145 ≤ Q2 <2.804	2.804 ≤ Q2 < 4.062	Q4 ≥ 4.062	*P*
Age (years)	13.219 (0.071)	13.396 (0.091)	13.585 (0.097)	13.725 (0.094)	<0.001
Sex (%)					<0.001
Male	62.554	50.439	45.230	47.430	
Female	37.446	49.561	54.770	52.570	
Race (%)					<0.001
Mexican American	7.441	10.865	14.546	18.027	
Non-Hispanic White	58.863	63.225	60.740	54.671	
Non-Hispanic Black	19.867	13.188	11.361	14.121	
Other Race	13.829	12.722	13.353	13.181	
Height (m)	1.585 (0.003)	1.566 (0.004)	1.572 (0.004)	1.591 (0.004)	<0.001
Waist circumference (m)	0.652 (0.002)	0.702 (0.002)	0.785 (0.002)	0.956 (0.003)	<0.001
SBP (mmHg)	104.300 (0.292)	105.039 (0.273)	107.243 (0.344)	110.607 (0.272)	<0.001
DBP (mmHg)	59.145 (0.373)	58.573 (0.392)	58.755 (0.381)	59.884 (0.398)	0.007
eGFR (ml/min/1.73m2)	134.797 (0.565)	129.354 (0.651)	130.392 (0.552)	133.242 (0.608)	<0.001
UACR (mg/g)	33.796 (3.271)	26.848 (2.138)	25.100 (3.182)	13.061 (0.754)	<0.001
ALT (U/L)	17.134 (0.193)	17.542 (0.240)	19.050 (0.395)	24.949 (0.553)	<0.001
AST (U/L)	24.618 (0.229)	23.654 (0.289)	23.078 (0.216)	24.287 (0.289)	<0.001
LDL-C (mmol/L)	2.241 (0.028)	2.291 (0.034)	2.428 (0.026)	2.557 (0.029)	<0.001
HDL-C (mmol/L)	1.432 (0.008)	1.406 (0.008)	1.311 (0.007)	1.173 (0.007)	<0.001
TC (mmol/L)	4.054 (0.018)	4.131 (0.020)	4.232 (0.020)	4.391 (0.020)	<0.001
CRP (mg/dL)	0.093 (0.012)	0.101 (0.015)	0.141 (0.008)	0.333 (0.015)	<0.001
Glycated haemoglobin (%)	5.153 (0.010)	5.157 (0.021)	5.135 (0.014)	5.194 (0.012)	<0.001
Albuminuria (%)	17.947	15.804	11.348	6.124	<0.001

Abbreviations: Q, quartile; SBP, systolic blood pressure; DBP, diastolic blood pressure; eGFR, estimated glomerular filtration rate; UACR, urine albumin-creatinine ratio; ALT, alanine aminotransferase; AST, aspartate aminotransferase; LDL-C, low–density lipoprotein cholesterol; HDL-C, high–density lipoprotein cholesterol; TC, total cholesterol; CRP, C-reactive protein.

### The association of BRI with albuminuria

The correlation between BRI and albuminuria risk among children and adolescents, as analyzed by weighted multivariate logistic regression, is presented in Table 2. In Model 1 (OR = 0.722, 95%CI: 0.684–0.762), BRI was significantly negatively correlated with albuminuria. Moreover, this negative association persisted significantly even after controlling for covariates in Models 2 (OR = 0.687, 95%CI: 0.648–0.729) and 3 (OR = 0.616, 95%CI: 0.526–0.722). Based on Model 3, with each increment in BRI, the risk of albuminuria is reduced by 38.4%, indicating that a higher BRI might decrease the likelihood of albuminuria.

**Table 2:** Association between Body Roundness Index and albuminuria.

Exposure	Model 1 OR(95%CI) *P* value	Model 2 OR(95%CI) *P* value	Model 3 OR(95%CI) *P* value
BRI (continuous)	0.722 (0.684, 0.762) <0.001	0.687 (0.648, 0.729) <0.001	0.616 (0.526, 0.722) <0.001
BRI (quartile)			
Quartile 1	reference	reference	reference
Quartile 2	0.812 (0.678, 0.972) 0.026	0.708 (0.590,0.850) <0.001	0.453 (0.332, 0.619) <0.001
Quartile 3	0.585 (0.489, 0.701) <0.001	0.484 (0.403,0.581) <0.001	0.282 (0.188, 0.422) <0.001
Quartile 4	0.298 (0.241, 0.369) <0.001	0.252 (0.203, 0.312) <0.001	0.180 (0.108, 0.300) <0.001
*P* for trend	<0.001	<0.001	<0.001

Model 1: Covariates were not adjusted.

Model 2: Age, gender and race were adjusted.

Model 3: Age, gender, ethnicity, ALT, AST, low-density lipoprotein cholesterol, high-density lipoprotein cholesterol, total cholesterol, C-reactive protein, estimated glomerular filtration rate (eGFR), glycated hemoglobin, systolic blood pressure, and diastolic blood pressure were adjusted.

Subsequently, we utilized weighted multivariate logistic regression analysis to investigate the relationship between quartiles of BRI and albuminuria (Table 2), with the lowest quartile serving as the reference. In models 1–3, compared to participants in the first quartile of BRI, patients with higher BRI levels had a significantly lower incidence of albuminuria, and the likelihood of albuminuria occurrence decreased with the rise in BRI (*P* for trend <0.001).

### Subgroup analysis

As depicted in Table 3, subgroup analyses were performed stratified by age, sex, and ethnicity to examine differences in the association between BRI and albuminuria across different subgroups. We found a significant negative correlation between BRI and albuminuria in all subgroups. Additionally, the OR value was found to be lower in males (OR: 0.525) compared to females (OR: 0.653), and significantly lower in the 8–14 years age group (OR: 0.490) compared to the 15–19 years age group (OR: 0.685), suggesting that each increase in BRI is linked to a greater decrease in the likelihood of albuminuria in males and younger individuals.

**Table 3:** Subgroup analysis of the association between Body Roundness Index and albuminuria.

Subgroup	Model 1 OR(95%CI) *P* value	Model 2 OR(95%CI) *P* value	Model 3 OR(95%CI) *P* value
Stratified by sex			
Male	0.621 (0.559, 0.690) <0.001	0.613 (0.550, 0.683) <0.001	0.525 (0.402, 0.684) <0.001
Female	0.719 (0.670, 0.773) <0.001	0.723 (0.672, 0.778) <0.001	0.653 (0.542, 0.788) <0.001
Stratified by ethnicity			
Mexican American	0.767 (0.695, 0.845) <0.001	0.750 (0.677, 0.830) <0.001	0.617 (0.514, 0.740) <0.001
Non-Hispanic White	0.683 (0.628, 0.743) <0.001	0.654 (0.598, 0.716) <0.001	0.604 (0.470, 0.775) <0.001
Non-Hispanic Black	0.756 (0.702, 0.814) <0.001	0.709 (0.654, 0.767) <0.001	0.665 (0.558, 0.793) <0.001
Other Race	0.768 (0.658, 0.897) 0.001	0.738 (0.626, 0.870) <0.001	0.622 (0.429, 0.902) 0.015
Stratified by age			
8–14	0.681 (0.634, 0.731) <0.001	0.649 (0.602, 0.699) <0.001	0.490 (0.384, 0.624) <0.001
15–19	0.789 (0.726, 0.859) <0.001	0.743 (0.677, 0.816) <0.001	0.685 (0.575, 0.816) <0.001

Model 1: Covariates were not adjusted.

Model 2: Age, gender and race were adjusted.

Model 3: Age, gender, ethnicity, ALT, AST, low-density lipoprotein cholesterol, high-density lipoprotein cholesterol, total cholesterol, C-reactive protein, estimated glomerular filtration rate (eGFR), glycated hemoglobin, systolic blood pressure, and diastolic blood pressure were adjusted.

In the subgroup analysis stratified by sex, ethnicity, or age, the model is not adjusted for the stratification variable itself.

### The association of BRI with UACR

Weighted linear regression analysis was used to verify the relationship between BRI and UACR (Table 4). In both the crude model and the adjusted model, there is a significant negative correlation between BRI and UACR. In Model 3, we found that an increase of 1 in BRI corresponded to a decrease of 5.424 mg/g in UACR (β: -5.424, 95%CI: -7.416 to -3.433). Subsequently, we further investigated the relationship between the quartiles of BRI and UACR. We found that the UACR of individuals in the highest quartile of BRI significantly decreased compared to those in the lowest quartile (β: -24.012, 95%CI: -37.440 to -10.583). Moreover, there was a significant trend indicating that as BRI increased, UACR decreased (*P* for trend <0.001).

**Table 4:** Association between Body Roundness Index and urine albumin-creatinine ratio.

	Model 1β (95%CI) *P* value	Model 2β (95%CI) *P* value	Model 3β (95%CI) *P* value
BRI(continuous)	-4.712 (-5.765, -3.658) <0.001	-5.235 (-6.324, -4.146) <0.001	-5.424 (-7.416, -3.433) <0.001
BRI (quartile)			
Quartile 1	reference	reference	reference
Quartile 2	-6.948 (-14.665, 0.768) 0.081	-9.285 (-17.276, -1.295) 0.025	-10.074 (-25.376, 5.228) 0.201
Quartile 3	-8.696 (-16.816, -0.576) 0.039	-11.996 (-20.342, -3.649) 0.006	-19.060 (-34.716, -3.403) 0.020
Quartile 4	-20.735 (-27.266, -14.204) <0.001	-23.689 (-30.580, -16.799) <0.001	-24.012 (-37.440, -10.583) <0.001
*P* for trend	<0.001	<0.001	<0.001

Model 1: Covariates were not adjusted.

Model 2: Age, gender and race were adjusted.

Model 3: Age, gender, ethnicity, ALT, AST, low-density lipoprotein cholesterol, high-density lipoprotein cholesterol, total cholesterol, C-reactive protein, estimated glomerular filtration rate (eGFR), glycated hemoglobin, systolic blood pressure, and diastolic blood pressure were adjusted.

### Analysis of the Non-linear relationship and saturation effect between BRI and albuminuria

**Fig 2:**
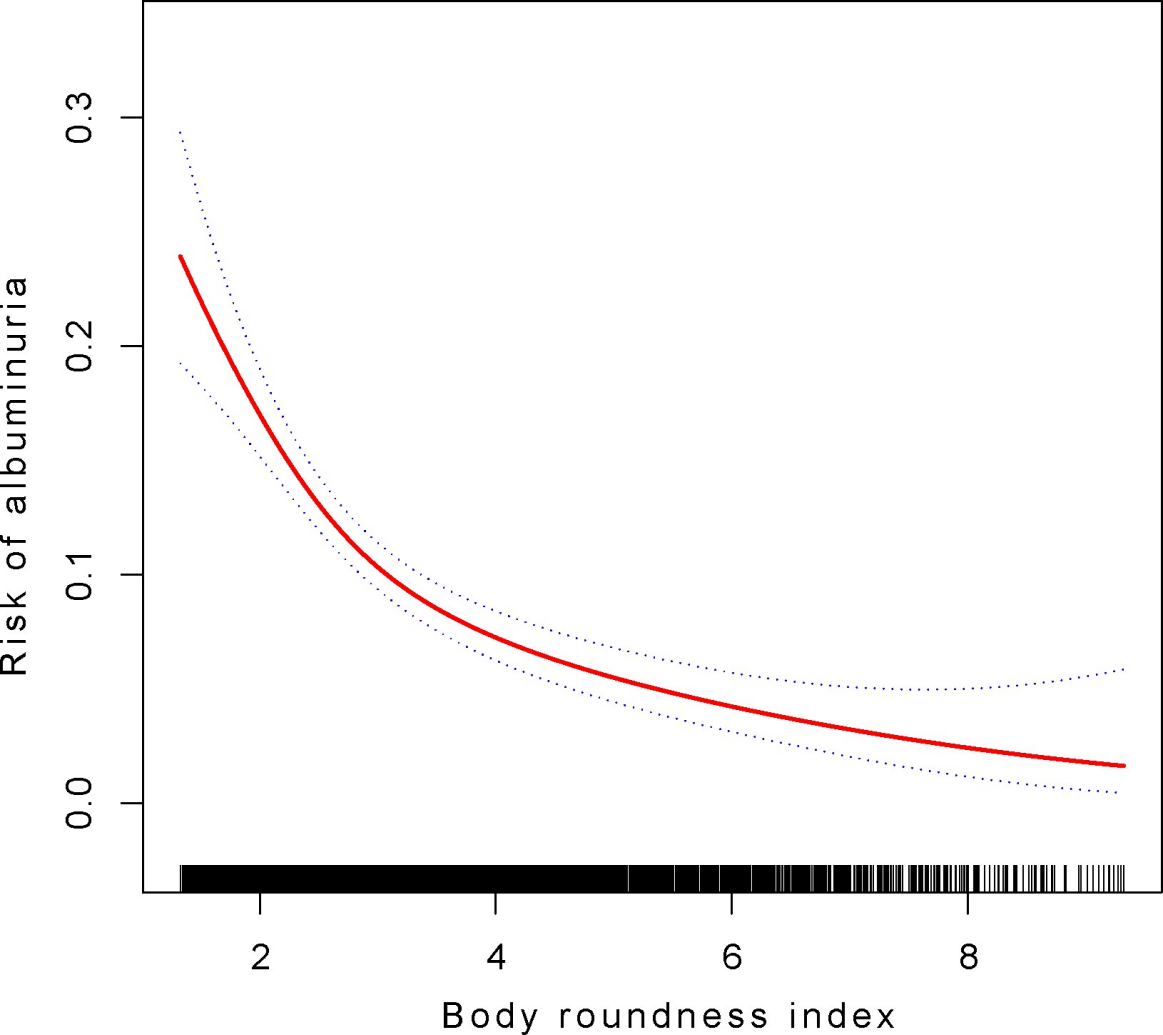
The nonlinear association between body roundness index and albuminuria. Through generalized additive model, we discovered a non-linear association between BRI and albuminuria. The red line in the model represents a smooth curve fit, and its 95% confidence interval is indicated by the blue line. And age, sex, race, systolic and diastolic blood pressure, as well as laboratory tests including ALT, AST, glycated hemoglobin, estimated glomerular filtration rate (eGFR), low–density lipoprotein cholesterol, high–density lipoprotein cholesterol, total cholesterol, and C-reactive protein were adjusted.

Through our employment of a smooth curve fitting approach, we discovered a non-linear correlation between BRI and the likelihood of albuminuria (as shown in Fig 2). In the subsequent log-likelihood ratio test, the *P*-value below 0.05 was observed (Table 5), which indicated a nonlinear association between BRI and albuminuria risk, necessitating the use of a two-piecewise linear regression model for fitting. Furthermore, through recursive algorithm and threshold effect analysis, we determined a BRI inflection point at 2.906 (Table 5). This suggests that for BRI values below 2.906, every increment in BRI corresponds to a 53.7% reduction in albuminuria risk (OR: 0.463, 95%CI: 0.359–0.596); however, for BRI values above 2.906, each increase only results in a 23.2% risk reduction (OR: 0.768, 95%CI: 0.673–0.877).

**Table 5:** The results of the two-piecewise linear regression model.

Fitting by the two-piecewise linear regression model	OR (95%CI)	*P* value
BRI Inflection point (2.906)		
BRI <2.906	0.463 (0.359, 0.596)	<0.001
BRI >2.906	0.768 (0.673, 0.877)	<0.001
Log-likelihood ratio test		0.002

The covariates employed in this model were identical to those depicted in Fig 2.

## Discussion

The cross-sectional study we conducted revealed a negative correlation between BRI and albuminuria risk. Further analysis using smooth curve fitting and log-likelihood ratio tests validated the nonlinear relationship, with a turning point for BRI identified at 2.906. However, the observed negative correlation should not be misinterpreted as an endorsement of excessively high BRI values. Excessive BRI values can lead to increased health risks. Moreover, based on our research results, there is a turning point for BRI values, beyond which the benefits of reducing albuminuria diminish. We suggest that maintaining BRI values within a moderate range may help reduce the incidence of albuminuria. Nevertheless, caution must be exercised to avoid excessively high BRI values due to associated health risks.

It is worth noting that the mechanisms behind this subtle relationship, as well as the optimal range for BRI, are still unclear. Moving forward, we recommend clinicians to consider incorporating BRI monitoring into routine health examination for children and adolescents. More research is necessary to explore the related mechanisms and establish the ideal BRI range that reduces the incidence of albuminuria without increasing the risk of obesity-related complications. Furthermore, concerted efforts towards advancing research can contribute to the body management of children and adolescents, improving public health outcomes.

While many studies have confirmed a positive link between obesity and albuminuria in adults, the relationship between these two factors among children and adolescents remains controversial. In Brazil, a cross-sectional research carried out with 64 overweight and obese individuals aged 5 to 19, revealed no significant correlation between the Z score for BMI and albuminuria [25]. However, differing from these results, our research, along with some others, has observed a negative correlation between obesity and albuminuria among children and adolescents. Evidence from a Dutch study involving a sample of 12-year-old children showed a significant negative relationship between urinary albumin concentration and z-BMI (β = -0.08, *p*-value = 0.013) [15]. Similarly, an Australian cross-sectional study with a sample of 975 children also reported a negative correlation between albuminuria and overweight or obesity [22]. Moreover, a prospective cohort study by Kim et al., involving 3418 children, discovered that the risk of albuminuria in children decreased as the BMI standard deviation score increased [26].

In adults, a positive relationship has been confirmed between obesity and albuminuria. This correlation is associated with renal microvascular damage, which is caused by hemodynamic alterations and mechanisms related to adipose tissue. Obesity can accelerate the progression of hypertension, which subsequently increases intraglomerular capillary pressure, promoting glomerular hyperfiltration and renal microvascular harm [27]. Moreover, obesity triggers the release of excessive inflammatory cytokines like tumor necrosis factor ɑ (TNF-ɑ) and leptin from adipose tissue, further aggravating glomerular injury [13]. Research in recent years has demonstrated the important role of insulin resistance in obesity-related chronic kidney disease. Evidence from animal studies has confirmed that obesity can induce a condition known as insulin resistance, characterized by the cells’ inability to respond effectively to insulin [28]. Insulin resistance can, in turn, exacerbate the development of renal fibrosis [29].

As previously mentioned, hypertension and inflammation caused by obesity are crucial in promoting the development of albuminuria in adults. Despite our observation that individuals in the higher quartile of BRI had higher blood pressure and elevated levels of C-reactive protein (Table 1), the observed negative correlation between BRI and albuminuria is puzzling. Hence, the mechanism explaining the inverse association between obesity and albuminuria among children and adolescents remains unknown and potentially complex. We proposed that age or hormonal alterations during puberty could be a key factor, or it could be due to an early compensatory stage, as indicated by the lower OR values in subgroups of younger ages compared to those of older ages. And this speculation needs to be investigated through prospective studies and fundamental research.

There are several advantages to our research. Firstly, as far as we are aware, this is the first study on the relationship between BRI and albuminuria, utilizing a novel body measurement index to gain deeper insights into the relationship between obesity and albuminuria. Secondly, we utilized a nationally representative sample and took into account the weights, making this study represent the multiethnic adolescent population in the United States. Furthermore, with a large sample size in our research, our results gain more credibility. We also discovered a nonlinear association between BRI and albuminuria via smooth curve fitting and determined the turning point. However, our study also has limitations. First, the cross-sectional design of our study did not allow for the confirmation of a causal relationship between BRI and albuminuria. Secondly, despite our best efforts to adjust for potential confounders, we cannot completely eliminate all confounding factors.

## Conclusion

Our research revealed a negative correlation between BRI and albuminuria among the pediatric and adolescent populations. Through the establishment of a smooth fitting curve, we discovered a non-linear association, and further threshold effect analysis identified the BRI inflection point. These findings from our research suggest that for children and adolescents, sustaining a moderate BRI might be beneficial in terms of decreasing the occurrence of albuminuria. However, additional research is paramount to thoroughly understand the relationship and mechanisms, and to define the suitable BRI range to prevent the detrimental effects of elevated BRI on other aspects of health.
